# Exploring the Possible Link between the Gut Microbiome and Fat Deposition in Pigs

**DOI:** 10.1155/2022/1098892

**Published:** 2022-01-22

**Authors:** Guangmin Zhao, Yun Xiang, Xiaoli Wang, Bing Dai, Xiaojun Zhang, Lingyan Ma, Hua Yang, Wentao Lyu

**Affiliations:** ^1^State Key Laboratory for Managing Biotic and Chemical Threats to the Quality and Safety of Agro-Products, Institute of Agro-Product Safety and Nutrition, Zhejiang Academy of Agricultural Sciences, Hangzhou 310021, China; ^2^College of Animal Science, Zhejiang A&F University, Hangzhou 311300, China; ^3^Institute of Animal Husbandry and Veterinary Medicine, Jinhua Academy of Agricultural Sciences, Jinhua 321011, China

## Abstract

Excessive lipid accumulation and high oxidative stress have become a serious health and economic problem in the pig industry. Fatness characteristics are crucial in pig production since they are closely related to meat quality. The gut microbiome is well acknowledged as a key element in fat deposition. But the link between gut microbiota and fat accumulation in pigs remains elusive. To examine whether there is a link between pigs' gut microbiome, lipogenic properties, and oxidative stress, we selected 5 high-fat pigs and 5 low-fat pigs from 60 250-day-old Jinhua pigs in the present study and collected the colon content, serum sample, and liver and abdominal fat segments from each pig for metagenomic analysis, the oxidative stress assay, and RT-qPCR analysis, respectively. The backfat thickness and fat content of the longissimus dorsi muscle were considerably higher in the high-fat pigs than in the low-fat pigs (*P* < 0.05). An obvious difference in GSH-Px and MDA in the serum between the high- and low-fat pigs was observed. After RT-qPCR analysis, we found the gene expression of *ACC1* and *SREBP1* in the liver and *FAS*, *PPARγ*, and *LPL* in the abdominal fat were significantly higher in high-fat pigs than in low-fat pigs (*P* < 0.05). Additionally, metagenomic sequencing revealed that high-fat pigs had a higher abundance of Archaeal species with methanogenesis functions, leading to more-efficient fat deposition, while low-fat pigs had higher abundances of butyrate-producing bacteria species that improved the formation of SCFAs, especially butyrate, thus alleviating fat deposition in pigs. Furthermore, a total of 17 CAZyme families were identified to give significant enrichments in different fat phenotypes of pigs. This study would provide a detailed understanding of how the gut microbiome influences fat deposition in pigs, as well as a hint for improving growth performance and fatness traits by manipulating the gut microbiome.

## 1. Introduction

The gut microbiota is a complex and dynamic ecosystem composed of trillions of microorganisms living in the digestive tract and acting as a regulator and barrier for other metabolic organs [1]. It has been linked to the onset of metabolic disorders such as obesity and oxidative stress. A chronic inflammatory process such as oxidative stress and metabolic disorders may result from the alteration in the structure of gut microbiota [2].

Fatness traits are not only a characteristic of obesity and oxidation injury but also very important in pig production since they are linked to fattening features and meat quality. The accumulation of excessive fat in animals and humans has become an increasing threat to the animal production industry as well as human health, which would lead to obesity and could further set off many other health problems such as cardiovascular disease, arthritis, and dementia. Furthermore, lipid accumulation and impaired lipid metabolism are associated with pathophysiological phenotypes in pigs, which has turned into a severe economic and health problem in pig production [3].

Chronic inflammation in adipose tissue due to excessive fat accumulation would promote proinflammatory status and oxidative stress [4]. The substantial rise in the generation of free radicals in humans and animals that disrupts the antioxidation-oxidation equilibrium is referred to as oxidative stress [5]. A variety of illnesses have been reported to be pathologically caused by oxidative stress, which has been shown to be correlated with fat accumulation [4]. In pigs, a significant number of free radicals are produced because of oxidative damage, which leads to several diseases [6]. More importantly, the development of the pig industry would be hampered by oxidative stress due to oxidative stress directly limiting the growth performance and health of pigs [5]. Therefore, studying the oxidative stress in pigs is of great significance to pig production, as well as other animals and human health.

Similar to humans, the pig gut microbiota is also a huge, sophisticated, and dynamic microbial population with a variety of vital biological functions [7], including energy harvest, methane synthesis, and the synthesis of short-chain fatty acids (SCFAs) [8]. Pig fatness phenotypes are closely associated with the composition and diversity of the microbial community in the gastrointestinal gut tract. The composition of fatty acids in the adipose tissues and liver could be altered by the activities of the gut microbiota [9]. The Jinhua pig in Zhejiang Province, China, is characterized by its slow growth speed and high intramuscular fat content, which is considered an excellent model for studying fat deposition [10]. In the previous study, we compared the bacterial community structure of obese Jinhua pigs and lean Landrace pigs and illustrated a clear causal connection between gut microbiota and fat deposition by fecal microbiota transplantation [11].

Most of the related studies focused on the differences in the structure of the intestinal microflora and fat deposition among different pig species. However, the relationship between fat-related phenotypes and the gut microbiome in pigs is still unclear. Therefore, in this study, fatness characteristics, fat-related gene expression in abdominal fat and liver, and oxidative stress in serum were measured in high- and low-fat pigs, respectively. Furthermore, the composition, diversity, and potential functions of the gut microbiota between the two groups were studied using a high-throughput metagenomic sequencing technique, further analyzing the association between the gut microbiome and fat deposition. This study will provide basic data for improving fatness traits in pig production by manipulating the gut microbiome.

## 2. Materials and Methods

### 2.1. Animal Experiments and Sample Collection

A total of 60 newly born Jinhua pigs were fed in 6 pens, 10 pigs per pen, in a commercial pig facility with a standard corn-soybean-based diet and water *ad libitum* as described previously [12]. Within two months before slaughter, each pig was healthy and had not been treated with antibiotics. At 250 days of age, pigs were killed to acquire blood samples, liver segments, and abdominal adipose tissue. Carcass traits, namely, body weight, loin muscle area, backfat thickness, and fat content in longissimus dorsi muscle, were analyzed by a principal component analysis (PCA). These statistics were utilized to determine which pigs were the most extreme for the selection of the most extreme pigs [13, 14]. Five high-fat (H) and five low-fat (L) pigs were chosen to collect luminal samples from the same colon location. Briefly, the gastrointestinal tract was peeled from the enterocoelia. The luminal samples were collected from the middle section of each pig's colon. Within 30 min after slaughter, all of the luminal samples were taken and divided into two parts: one was for the measurement of SCFAs, and the other one was placed in a -80°C refrigerator until DNA extraction after being dipped in liquid nitrogen.

All the procedures for animal experiments were approved by the Zhejiang Academy of Agricultural Sciences Institutional Animal Care strictly according to the relevant rules and regulations (Ethic code: ZAAS-2017-009).

### 2.2. Luminal DNA Extraction, Metagenomic Sequencing

According to the manufacturer's recommendations, the QIAamp Fast DNA Stool Mini Kit (Qiagen, Germany) was used to extract the luminal DNA from each colon content sample. A NanoDrop 1000 spectrophotometer (NanoDrop Technologies, Wilmington, DE, USA) was used to measure the amount and quality of DNA, and sterile water was used to dilute the DNA concentration to a final concentration of 1 ng/L. The Illumina TruSeq™ DNA Sample Prep Kit was used to generate libraries, which were sequenced on an Illumina HiSeq 2500 platform by a commercial sequencing company, Novogene (Beijing, China).

### 2.3. De Novo Assembly of Short Reads

We used trimmomatic [15] for the quality control of raw datasets to remove the 3′- and 5′-end of reads, eliminate poor quality bases (<20), and trim containing 10% N of reads, and small segments (<75 bp). The BWA [16] was performed to align the reads with the pig genome to filter out the host DNA from the reads. Megahit [17] (https://github.com/voutcn/megahit) was used to de novo assemble the filtered reads for each sample. Contigs were continuous sequences that had clear linkages between each other.

### 2.4. Gene Prediction and Functional Annotation

The ORFs (open reading frames) from each sample's contigs were predicted using METAProdigal (http://prodigal.ornl.gov/). We used cd-hit [18] software (http://www.bioinformatics.org/cd-hit/) to exclude the redundant genes (parameters for 95% identity; 90% coverage) from all the predicted ORFs. And then, Salmon [19] (https://github.com/COMBINE-lab/salmon) was used to determine gene abundances by mapping the original sequences to anticipated genes. Finally, with BLASTP [20] (BLAST Version 2.2.28+, http://blast.ncbi.nlm.nih.gov/Blast.cgi), the taxonomy of the colon microbiota was evaluated against the NR database. The relative abundances of taxa at the domain, phylum, genus, and species levels were determined using taxonomic profiles. The NMDS was conducted at the gene level based on the Bray-Curtis dissimilarity matrices. The KEGG database (http://www.genome.jp/kegg/) was used to annotate the contigs with a BLAST *E* value of 1*e* − 5. Hmmscan (http://hmmer.janelia.org/search/hmmscan) was used to annotate the CAZy functions.

### 2.5. Real-Time Quantitative PCR (RT-qPCR)

Gene expression was measured in the liver and abdominal adipose tissue. According to the manufacturer's instructions, the RNeasy Plus Mini Kit (Qiagen) was used to isolate the total RNA from each sample of abdominal fat and liver segments. The first-strand cDNA was generated using the SuperScript II Reverse Transcription Kit (Invitrogen). The gene expression was evaluated by RT-qPCR on the ABI Prism 7700 Sequence Detector (Applied Biosystems) with gene-specific primers (see Supplementary Tables S1). The following were the reaction conditions: 95°C for 5 min, followed by 40 cycles of 15 s at 94°C, 30 s at 63°C, and 1 min at 72°C and fluorescence collection as previously described [11]. The relative gene expression level was determined by the 2^-∆∆Ct^ method [21] using the geometric mean of glyceraldehyde 3-phosphate dehydrogenase (GAPDH) mRNA as the housekeeping gene for the data normalization.

### 2.6. Colonic Butyrate-Producing Functional Gene Analysis

The qPCR was performed in triplicate for the DNA extracted from each sample to assess the gene copy number of the butyrate-producing functional genes, namely, butyrate kinase and butyryl CoA: acetate CoA transferase, in pig intestinal contents on an ABI Prism 7700 Sequence Detector (Applied Biosystems) [22, 23] using gene-specific primers (see Supplementary Tables S2) and SYBR Green PCR Master Mix (Takara, Tokyo, Japan). The thermal cycling system was 95°C for 2 min, 35 cycles of 15 s at 95°C, 45 s at 58°C, and 1 min at 72°C. The specificity of the reaction for each gene was verified by a melting curve analysis. Standard curves from known quantities of plasmid DNA were used to determine the copy number of each gene. The data from qPCR experiments was represented as gene copies per gram of luminal content.

### 2.7. Oxidative Stress Determination

Serum antioxidant levels were determined after homogenization with saline (1 : 9 *w*/*v*) followed by centrifugation at 11,000 × g for 15 minutes at 4°C. As recommended by the manufacturer's instructions, we used Nanjing Jiancheng Bio (Nanjing, China) diagnostic kits to detect indicators including malondialdehyde (MDA), superoxide dismutase (SOD), and glutathione peroxidase (GSH-Px).

### 2.8. SCFA Measurement

As mentioned in our previous study [24], the gas chromatographic (GC) was used to determine the levels of SCFAs in each luminal content sample. Shortly, the 100 mg luminal content sample was weighed into a 1.5 mL centrifuge tube and suspended in 9 volumes of Milli-Q water. Following a 10 min centrifugation at 10,000 rpm, 1,000 *μ*L of the supernatant was supplemented with 0.2 mL of crotonic acid (internal standard). Finally, following the membrane filtering (0.22 *μ*m), the mixture was put into the GC-2010 plus (Shimadzu, Kyoto, Japan) with an FID detector operating at 180°C. The chromatographic conditions were as follows: column 110°C, vaporization chamber 180°C. The carrier gas was nitrogen at 0.06 MPa while the auxiliary gas was hydrogen and air with the pressure of 0.05 MPa and 0.05 MPa, respectively.

### 2.9. Statistical Analysis

All statistical analyses were conducted using the unpaired two-tailed Students' *t*-test with a *P* value < 0.05 as the level of statistical significance. Data were presented as mean ± standard deviation (SD).

## 3. Results

### 3.1. Fatness Phenotypes between the High- and Low-Fat Pigs

**Figure 1:**
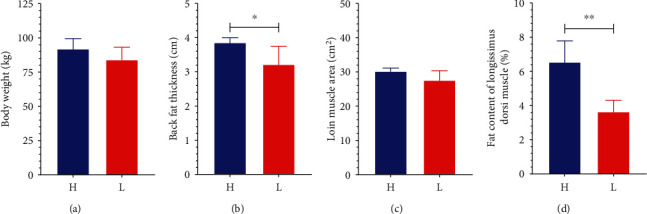
Fatness phenotypes in the high- and low-fat pigs. Newly born piglets were fed commercial feed for 250 days before being sacrificed. The body weight (a), backfat thickness (b), loin muscle area (c), and fat content of longissimus dorsi muscle (d) were determined. Data are expressed as mean ± SD (*n* = 5) and analyzed by the unpaired two-tailed Students' *t*-test. H: the high-fat pigs; L: the low-fat pigs. ^∗^*P* < 0.05 and ^∗∗^*P* < 0.01.

To determine whether there is a significant difference in the fatness phenotypes between the two groups, pigs were raised under standard management and sacrificed at 250 days old to determine body weight, loin muscle area, backfat thickness, and fat content of longissimus dorsi muscle. The high-fat pigs showed significantly higher backfat thickness and fat content of longissimus dorsi muscle than the low-fat pigs (*P* < 0.05, see Figures 1(b) and 1(d)). However, the two groups had no significant differences in body weight and loin muscle area (see Figures 1(a) and 1(c)).

### 3.2. Expression of Lipid Metabolism Genes

**Figure 2:**
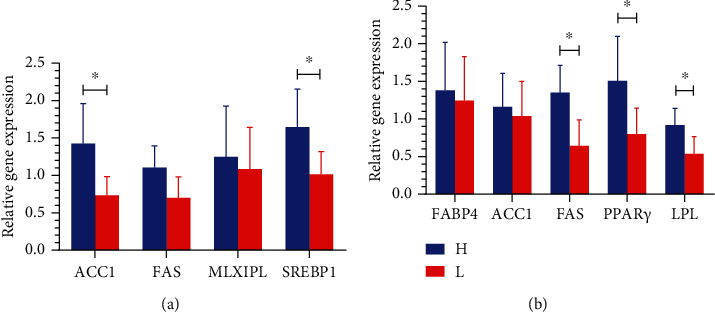
Relative gene expression levels of lipid metabolism genes in the liver (a) and abdominal fat (b) of Jinhua pigs. The tissue segments of liver and abdominal fat were collected from the high- and low-fat pigs at 250 days old for RNA isolation. The relative expression level of each indicated gene was determined by RT-qPCR using the 2^-*ΔΔ*Ct^ method. Data normalization employs GAPDH as a housekeeping gene. Data are expressed as mean ± SD (*n* = 5) and analyzed by the unpaired two-tailed Students' *t*-test. H: the high-fat pigs; L: the low-fat pigs. ^∗^*P* < 0.05.

To further test the gene expression of lipid metabolism in the liver and abdominal fat tissue, we collected liver and abdominal fat segments from each of the high- and low-fat pigs, followed by RNA isolation and RT-qPCR analysis. In the liver, the two key lipogenic genes, *ACC1* (acetyl-CoA carboxylase-1) and *FAS* (fatty acid synthase), showed higher levels in the high-fat pigs than in the low-fat pigs, with *ACC1* being significantly higher (*P* < 0.05, see Figure 2(a)). Additionally, *SREBP1* (Sterol Regulatory Element Binding Protein-1) was also significantly higher in the liver of high-fat pigs (*P* < 0.05), while the gene expression of *MLXIPL* (Mlx-interacting protein-like) showed no significant difference (see Figure 2(a)).

In the abdominal fat tissue, a significant increase in the gene expression of lipogenesis genes, *FAS* and *LPL* (lipoprotein lipase), and the adipogenesis gene *PPARγ* (peroxisome proliferator-activated receptor-*γ*) in the high-fat pigs was observed (*P* < 0.05). The *ACC1* and *FABP4* (fat acid-binding proteins 4) showed higher levels in the high-fat pigs than the low-fat pigs with no significant difference (*P* > 0.05, see Figure 2(b)).

### 3.3. Oxidative Stress Levels in Pigs with Different Fat Deposition

**Figure 3:**
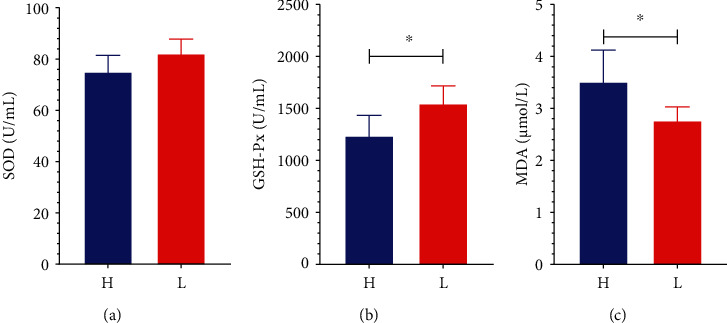
Oxidative stress levels in the high- and low-fat pigs. The serum samples were collected from the high- and low-fat pigs at 250 days old for the determination of SOD activity (a), GSH-Px activity (b), and MDA content (c) by different assays. Data are expressed as mean ± SD (*n* = 5) and analyzed by the unpaired two-tailed Students' *t*-test. H: the high-fat pigs; L: the low-fat pigs. ^∗^*P* < 0.05.

To evaluate whether oxidative stress functions in the lipid metabolism, the key markers of oxidative stress, including MDA, GSH-Px, and SOD, were further analyzed. The low-fat pigs had decreased MDA content and increased GSH-Px activity compared to the high-fat pigs (see Figure 3, *P* < 0.05).

### 3.4. Profiling of the Colon Metagenome in Pigs

Next, the composition and diversity of the colonic microbiota between the two groups were investigated by metagenomic sequencing. For the construction of libraries, we pooled 10 samples of luminal DNA from Jinhua pigs. Each DNA pool included an average of 12.84 Gb of raw data (9.94–19.00 Gb). Clean data ranged from 9.33 Gb to 17.90 Gb after removing low-quality reads and host contamination. All short sequence data was assembled by Megahit [25] (https://github.com/voutcn/megahit). Ten DNA pool samples had a total number of contigs ranging from 243,029 to 710,018 with a length greater than 500 bp. METAProdigal [26] (http://prodigal.ornl.gov/) predicted the open reading frame (ORF) of each contig. A total of 5,617,408 ORFs that were longer than 60 bp were obtained in the pool of ten samples (see Table 1).

### 3.5. Comparison of Microbial Domains between High- and Low-Fat Pigs

**Figure 4:**
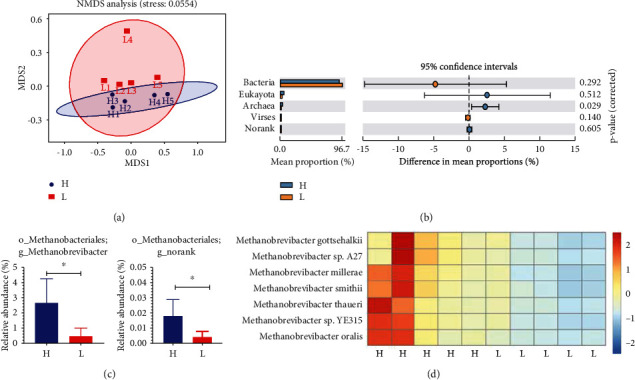
Microbial profiles of the high- and low-fat pigs. The colon content samples were collected at 250 days old for DNA isolation and metagenome sequencing. (a) NMDS analysis of colon content samples was performed with the abundance of genes. (b) The relative abundance of microbial domains between the two groups. (c) The relative abundance of Archaea at genus level between the two groups. ^∗^*P* < 0.05. (d) The relative abundance of Archaea at species level between the two groups. The heatmap was generated with *z*-score calculated from the relative abundance of each Archaea species. Data are expressed as mean ± SD (*n* = 5) and analyzed by the unpaired two-tailed Students' *t*-test. H: the high-fat pigs; L: the low-fat pigs.

Nonmetric multidimensional scaling (NMDS) revealed a robust separation between high- and low-fat pigs (see Figure 4(a)). The microbial structure in the colons of the two groups was compared. The relative abundance of Archaea in the high-fat group was obviously higher than that of low-fat pigs (*P* < 0.05) while eukaryota and viruses were not significantly different (*P* > 0.05, see Figure 4(b)). We then compared the relative abundance of Archaea bacterial genera between the colon microbiomes of the two groups with distinct fatness. Two genera (see Figure 4(c)) and seven species (see Figure 4(d)) were identified to give a significant difference in the relative abundances between the two groups (*P* < 0.05), which all belong to the methanogen genus.

### 3.6. Significant Difference in Bacterial Community Compositions of Pigs with Distinct Fatness Phenotypes

**Figure 5:**
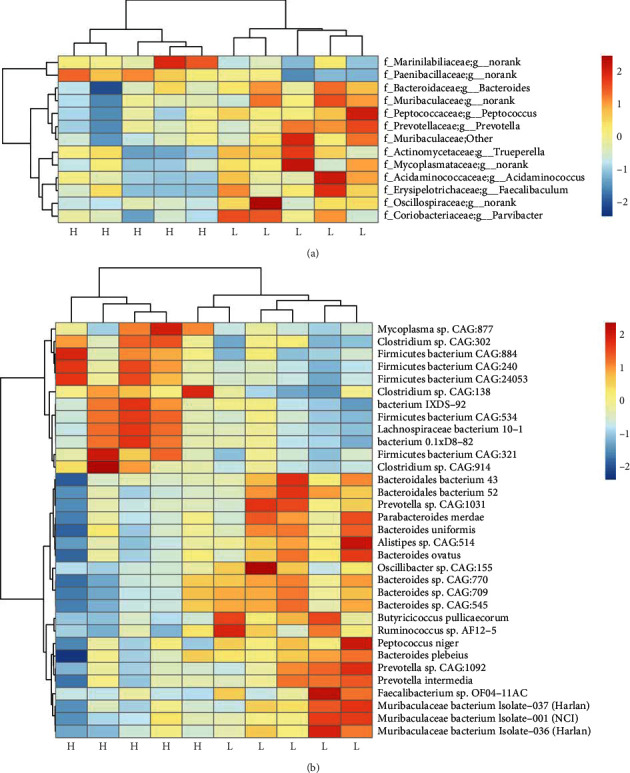
The relative abundance of microbiota in the colon of the high- and low-fat pigs. The colon content samples were collected from 5 high-fat and 5 low-fat pigs at 250 days old for DNA isolation and metagenome sequencing. The genus (a) and species (b) levels of bacteria in the colons of high- and low-fat pigs were compared. Heatmaps were generated with *z*-score calculated from the relative abundance of each bacteria genus (a) or species (b). The top 13 genera and 33 species are shown. H: the high-fat pigs; L: the low-fat pigs.

As shown in Supplementary Figure S1, the low-fat pigs exhibited a higher *α* diversity of gut microbiota than the high-fat pigs. Further studies were performed on the microbial community structures between the two groups with distinct fatness phenotypes in the colon. *Bacteroidetes* were found in significantly higher abundance in the colons of low-fat pigs than in high-fat pigs (*P* < 0.05, Supplementary Figure 2). At the genus level, 13 genera were significantly different between the two groups. *Prevotella* and *Bacteroides* were significantly more abundant in the colons of low-fat pigs (*P* < 0.05). *Prevotella* relative abundance was 2.979% and 5.659% between the two groups, respectively, while *Bacteroides* relative abundance was 1.300% and 1.935%, respectively. Additionally, the relative abundance of *Prevotella* and *Bacteroides* was above 1.0%, whereas most other genera were below 1.0% in the colon. It is worth noting that *Acidaminococcus* was significantly enriched in the low-fat pigs (*P* < 0.05, see Figure 5(a)).

At the species level, we discovered 33 bacterial species giving different enrichments between the two groups. Among these species, 12 species were enriched in the high-fat pigs, while the other 21 species were more abundant in the low-fat pigs (see Figure 5(b)). Furthermore, we observed that ten of the 12 species with higher abundances in the high-fat pigs are *Firmicutes* and *Tenericutes*. The bacteria abundant in low-fat pigs have been correlated with fiber fermentation and butyrate production, namely, *Ruminococcus* sp. AF12-5 [27], *Faecalibacterium* sp. OF04-11AC [28], and *Oscillibacter* sp. CAG:155 [29].

### 3.7. Gut Microbiome Functional Capacity in Pigs with Different Fatness Phenotypes

**Figure 6:**
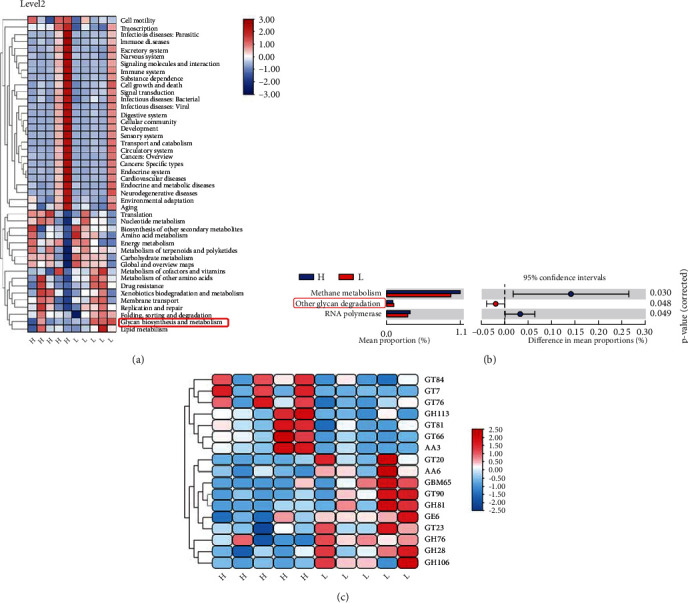
Enrichment of CAZymes and KEGG functions in the colon. The colon content samples were collected from 5 high-fat and 5 low-fat pigs at 250 days old for DNA isolation and metagenome sequencing. (a) The relative abundance of each KEGG pathway (Level 2) in the high- and low-fat pigs after an enrichment analysis. (b) The relative abundance of each KEGG subsystem (Level 3) in the high- and low-fat pigs after an enrichment analysis. (c) The relative abundance of each CAZyme functional term after an enrichment analysis. Heatmaps were generated with *z*-scores calculated from the relative abundance of each KEGG or CAZyme function. H: the high-fat pigs; L: the low-fat pigs; GH: Glycoside Hydrolase; GT: Glycosyl Transferase; PL: Polysaccharide Lyase; CE: carbohydrate esterases; CBM: carbohydrate-binding module; AA: Auxiliary Activities.

The functional capacity of the gut microbiome between the two groups was further analyzed using KEGG annotation. At Level 2 of KEGG function analysis, “Glycan biosynthesis and metabolism” was the most significantly different metabolic function between the two groups (see Figure 6(a)). At Level 3, “Methane metabolism,” “Other glycan degradation,” and “RNA polymerase” were found to be the significant markers in the pigs with a distinct fatness phenotype. More importantly, the relative abundance in the function pathway profile of “Other glycan degradation” was higher in the low-fat pigs than in the high-fat pigs (see Figure 6(b)).

To further study the colonic microbiota's functional capacity, we analyzed the enzymes that break down glycans (CAZymes: Carbohydrate-Active Enzymes) [30]. There were significant differences in the enrichments of 17 CAZyme families between the two groups of pigs (*P* < 0.05, see Figure 6(c)). We found 2 enriched CAZyme genes, which function to degrade carbohydrates such as cellulose, hemicellulose, and starch, while 6 were enriched in the low-fat pigs (GH28, GH76, GH81, GH106, CE6, and AA6). GTs had the function of carbohydrate synthesis. In the present study, the high-fat pigs had 5 enriched GTs, namely, GT7, GT66, GT76, GT81, and GT84, while the low-fat pigs had 3 enriched GTs, namely, GT20, GT23, and GT90. In addition, the carbohydrate-binding modules (CBMs) are noncatalytic CAZymes with the function of the breakdown of complex carbohydrates. The low-fat pigs had 1 enriched CBMs in the colon, CBM65.

### 3.8. Changes in Colonic SCFA Levels in the Pigs with Different Fatness Phenotypes

**Figure 7:**
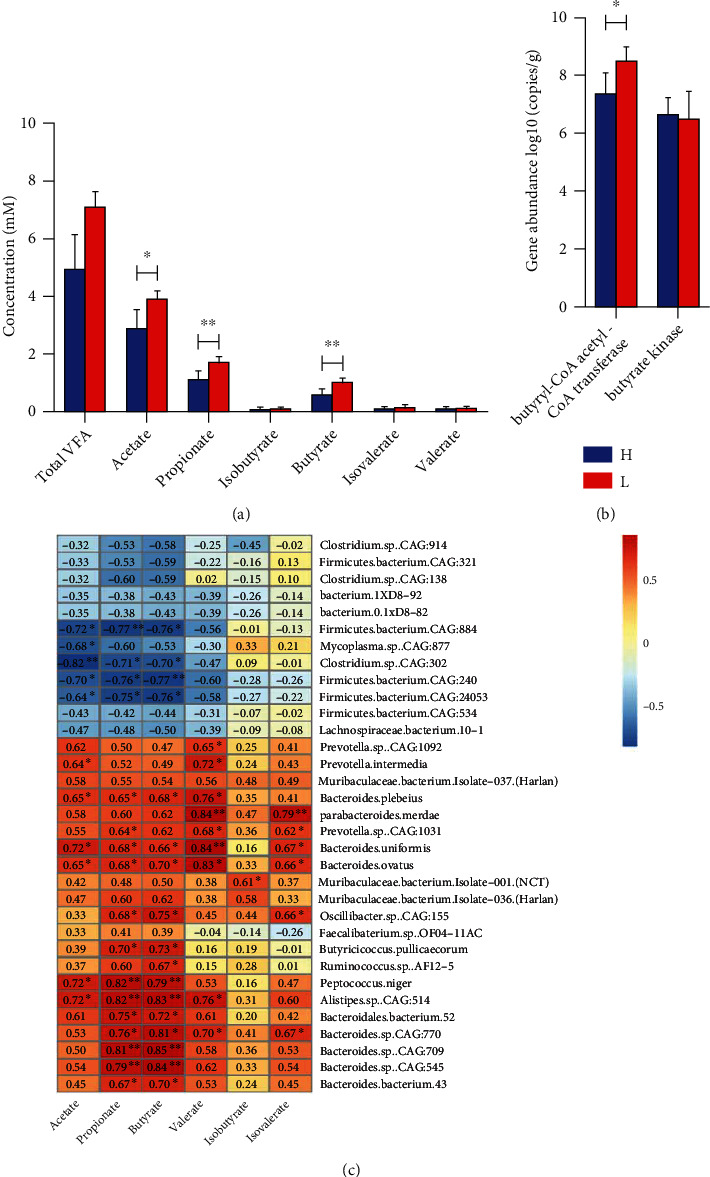
The association between colonic SCFA levels and colonic microbiome in the high- and low-fat pigs. The colon content samples were collected from 5 high-fat and 5 low-fat pigs at 250 days old for the determination of SCFA content, DNA isolation, and metagenome sequencing. (a) Concentrations of SCFAs in the colons of the two groups were examined by GC. Data are expressed as mean ± SD (*n* = 5) and analyzed by the unpaired two-tailed Students' *t*-test. (b) The gene abundance of the butyrate-producing genes in the colon of the high- and low-fat groups. Data was expressed as log10 gene copies of total DNA/g colon content. (c) The Spearman correlation between the 33 differentially abundant species and SCFAs in the colon of Jinhua pigs. The *X*-axis shows SCFAs, whereas the *Y*-axis shows bacteria species. The chart's various colors and numbers reflect the correlation coefficient between the SCFA and bacteria species indicated. H: the high-fat pigs; L: the low-fat pigs. ^∗^*P* < 0.05 and ^∗∗^*P* < 0.01.

To examine whether pigs with different fatness phenotypes would alter the SCFA content in the colon, we quantified the absolute concentrations of the total SCFAs, namely, propionate, acetate, and butyrate. In comparison to the high-fat pigs, the low-fat pigs' colons had much higher levels of acetate, propionate, and butyrate (see Figure 7(a)). Furthermore, the colon of low-fat pigs had a relatively higher abundance of butyryl-CoA acetate-CoA transferase (see Figure 7(b)), which functions to modulate the butyrate production in the colonic microbiome.

To reveal the potential linkage between gut microbiota and SCFAs, we correlated between differential gut microbiota and SCFA levels. At the species level, *Bacteroides plebeius*, *Bacteroides uniformis*, *Bacteroides ovatus*, *Peptococcus niger*, *Bacteroides* sp. *CAG:770*, *709*, *545*, and *Bacteroidales bacterium 43*, which belong to the Bacteroidetes phyla, were positively correlated with intestinal contents SCFA levels, especially with propionate and butyrate. Species *Firmicutes bacterium CAG:884*, *240*, *24053* and *Clostridium* sp. *CAG:302* (which belongs to phylum Firmicutes) were negatively correlated with acetate, propionate, and butyrate levels. These results revealed the changed composition and function of the gut microbiota might well have contributed to the SCFA production in the colon (see Figure 7(c)).

## 4. Discussion

The gut microbiota is considered essential in the utilization of nutrients and energy and the maintenance of healthy status in farm animals. Thus, the gut microbiota is regarded as an important component affecting the growth performance and development of pigs. Nowadays, numerous studies investigating the structure of the gut microbial community in pigs by metagenomics have been proposed to reveal the association between gut bacterial species and porcine fatness in different breeds [31]. We explored the interaction among the gut microbiota, lipogenic features, and oxidative stress in pigs with two different fat phenotypes. Furthermore, we tried to implicate the possible relationship between the gut microbiome and fat deposition in pigs using metagenomic analysis.

The liver and adipose tissue are the main parts of the body that deposit, metabolize, and transport fat. *ACC1*, *FAS*, *SREBP-1*, and *ChREBP* are the key genes in de novo fat synthesis [32]. The gene expression of *ACC1* and *SREBP-1* was significantly higher in the liver of the high-fat pigs than in the low-fat pigs, indicating that high-fat pigs might have a stronger ability to synthesize fat than low-fat pigs. The abdomen and intestines are the main sites of fat accumulation. The “master regulator” of adipogenesis is *PPARγ*, required for fat cell production [33]. The upregulation of *PPARγ*, *LPL*, and *FAS* in the high-fat pigs indicated enhanced lipid synthesis in the abdomen. Collectively, the imbalance in lipid metabolism might contribute to the abnormal fat accumulation in pigs.

On the other hand, the development of the pig industry would be hampered by oxidative stress due to oxidative stress directly limiting the growth performance and health of pigs. MDA is a biomarker for free radical species-related damage [34]. In the present study, MDA levels in serum were obviously higher in high-fat pigs than in low-fat pigs. Increased oxidative stress associated with fat consumption may alter the bacterial composition and the expression of lipogenic genes. GSH-Px is a critical enzyme that catalyzes hydrogen peroxide decomposition, which would protect the structure and function of the cell membrane [35]. GSH-Px levels were significantly lower in high-fat pigs. These findings show that high-fat pigs are more susceptible to oxidative stress than low-fat pigs, and hence more susceptible to obesity.

Consistent with our earlier investigation of 16S rRNA gene sequencing in pigs [23], the bacterial community of the two groups showed taxonomic discrepancies. It implies that bacteria contribute more to host fatness than other microbial kingdoms. Bacteria, not other microbial kingdoms, are responsible for the majority of the breakdown and fermentation of feed biopolymers [36]. Interestingly, the majority of the species with significantly increased abundances in the low-fat pigs were from the *Bacteroides* genus, which is one of the most frequent core genera in the pig colony. *Bacteroides* was able to utilize the fermentation of dietary fibers to produce acetate, propionate, and butyrate [24]. *Prevotella* and *Bacteroides* were found to be significantly less abundant in the colons of high-fat pigs than in low-fat pigs. The more *Bacteroides–Prevotella–Porphyromonas* there are in developing pigs, the better their ability to ferment polysaccharides to SCFAs [37]. Furthermore, *Bacteroides*-*Prevotella* was negatively correlated with inflammation and fat mass development in diet-induced obese mice [38]. Taken together, these results suggest that enhanced SCFA production in the low-fat pigs might be linked with the higher abundance of *Prevotella* and *Bacteroidetes*, which further alleviate host lipid accumulation.

Accordingly, our findings indicated that colonic SCFA levels were significantly higher in pigs with low-fat in comparison to pigs with high-fat traits. Butyrate could directly activate AMP kinase to prevent mice from having excess fat deposition in the liver [39]. The mice transplanted from lean cotwin's fecal microbiota showed lower fat storage in adipose tissue compared to the mice transplanted from obese cotwin's fecal microbiota [40]. Importantly, we found correlations between SCFAs and differential gut microbiota in pigs. The phylum *Firmicutes* and its subordinate species were related to reduced SCFAs, whereas the phylum *Bacteroidetes* and its subordinate species were related to increased SCFAs. All SCFAs showed synchronous correlations with the differential gut microbiota, suggesting that the differential gut microbiota and the SCFAs they produced might work synchronously in fat deposition.

Based on the metagenomics, KEGG analysis revealed the gut microbiome functional changes in the two groups. The metagenomes of the high-fat pigs had a significantly higher enrichment of methane metabolism and RNA polymerase. Methane metabolism, in particular, has been reported to produce methane from hydrogen with carbon dioxide, acetate, and various methyl metabolites [41]. Bacterial NADH dehydrogenases could be suppressed by the improvement of H_2_, resulting in a decrease in ATP output [42]. Methane is able to slow down the gut transport rate, which allows nutrients to stay in contact with the gut for longer, giving more time for nutrients and energy to be absorbed. This might explain why the high-fat pigs gained more weight than the low-fat pigs.

Since glycan degradation was found to be significantly enriched in the functional microbiome of low-fat pigs, we further determined that CAZymes shifted between the two groups in the present study. The results showed that GH, CE, PL, AA, and CBM, which exert a function of carbohydrate degradation, were enriched in low-fat pigs, implying that low-fat pigs might have a better ability to deconstruct complex substrates. For example, the colon of the low-fat pigs showed a higher relative abundance of GH28, GH76, GH81, and GH106, which are polygalacturonosidase [43], *α*-1,6-mannanase [44], *β*-1,3-glucanase [45], and *α*-L-rhamnosidase [46], respectively.

In contrast to the low-fat pigs, the higher abundance of GTs in the high-fat pigs showed that the colon microbiomes of high-fat pigs might have a better capability to utilize hydrolytic products to produce SCFAs, thus providing more energy to high-fat pigs. Generally speaking, feed-efficient animals generate more SCFAs and less methane [36]. The higher level of SCFAs and lower abundance of methanogenic functions in the colon of low-fat pigs suggest that low-fat pigs may be more feed-efficient than high-fat pigs. To confirm our assumptions, further research on feed efficiency and methane emissions is required in the future.

Notably, the higher relative abundance in genus *Methanobrevibacter* and various species, namely, *M. millerae*, *M. gottschalkii*, *M.* sp. *YE315*, *M. thaueri*, *M. smithii*, *M. sp. A27*, and *M. oralis*, was observed in the colons of high-fat animals, which suggests that more methane might be produced, resulting in more efficient fat deposition in the high-fat pigs [47]. The metagenome gave us a chance to study the gut microbiome at different kingdom levels, such as eukaryote and virus, except for bacteria and Archaea. The interactions between bacteria and eukaryotes or viruses might also change the status of host fat deposition, because our main concern was not with eukaryotes or viruses in the present study. Additional research in the future is needed.

**Figure 8:**
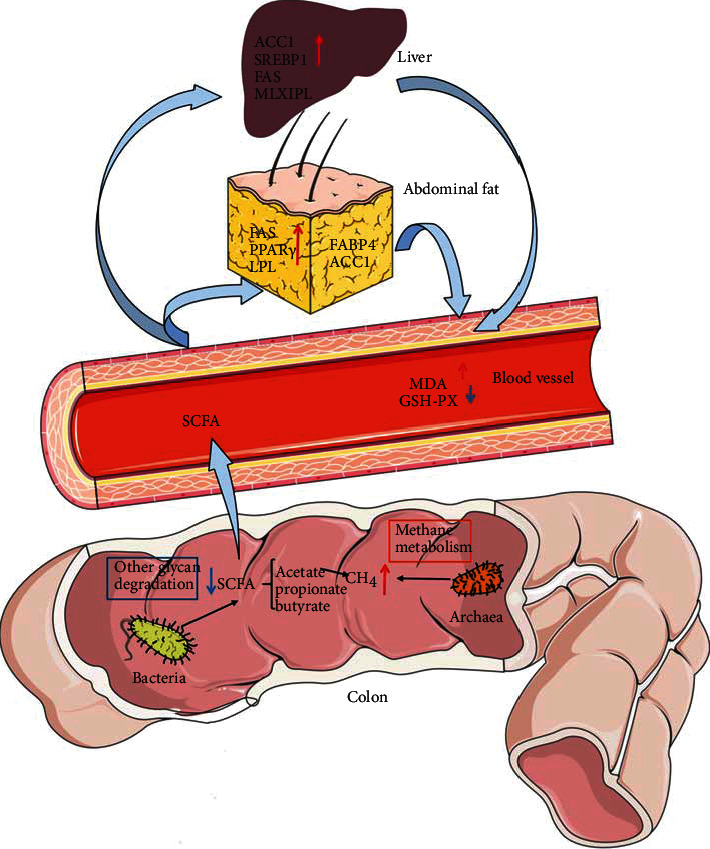
Relationship between intestinal microbiome and fat deposition.

In summary, we found that there were abundant methanogenic Archaea and relatively lower SCFA-producing bacteria in the colons of high-fat pigs compared to low-fat pigs. The increased methane produced by the Archaea and the decrease of SCFAs would promote fat deposition in the liver and abdomen, which is manifested by the high expression of adipogenic genes in the liver (*ACC1* and *SREBP1*) and abdominal fat (*FAS*, *PPARγ*, and *LPL*), furthering the oxidative stress injury (see Figure 8).

## 5. Conclusions

In summary, the high oxidative stress and lipid metabolism dysbiosis in the high-fat pigs could contribute to the fat deposition in Jinhua pigs. Host fat deposition is influenced by both the methanogenesis functions of Archaea and the short-chain fatty acids produced by bacteria. In addition, we discovered 17 CAZyme families and 3 KEGG pathways (Level 3) with distinct enrichments in the high-fat and low-fat pigs. The present study would give a deep insight into how gut microbiomes influence fat deposition in pigs and provide a hint for improving growth performance and fatness traits by manipulating gut microbiomes.

## Figures and Tables

**Table 1:** Data summary of metagenomics.

Sample	Raw bases (Mbp)	Clean bases (Mbp)	Contigs	Contigs bases (bp)	N50 (bp)	ORFs	Average length (bp)
H1	11,300	10,500	277327	345242102	1,337	581,709	619.16
H2	9,940	9,330	243029	303872492	1,315	520,091	603.23
H3	10,300	9,570	249525	280357289	1,151	448,785	619.48
H4	12,400	11,500	298036	321492268	1,026	444,650	574.05
H5	15,000	13,800	710018	626251331	824	526,644	610.65
L1	12,500	11,700	304407	394804127	1,379	797,874	629.86
L2	10,500	9,980	285676	351527843	1,283	586,934	636.42
L3	19,000	17,900	399192	555397511	1,523	458,592	539.68
L4	14,200	13,500	275692	415245171	1,805	492,082	504.58
L5	13,300	12,100	351437	358387739	942	760,047	372.78

## Data Availability

The colon metagenome sequences were deposited into NCBI Sequence Read Archive (SRA) under the accession number PRJNA760651 (http://www.ncbi.nlm.nih.gov/bioproject/760651).

## Supplementary Materials

Supplementary Materials Figure S1: *α* diversity (Shannon index) of the gene, genus, KEGG orthologs (KO), and eggNOG (OG) functions. Figure S2: comparison of abundance of Bacteroidetes in high-fat and low-fat pigs. Supplementary Table S1-S2: sequences of the primers used in this study.
