# Supplementary material for: Exploring the Possible Link between the Gut Microbiome and Fat Deposition in Pigs
Source: Oxid Med Cell Longev. 2022 Jan 22;2022:1098892. doi: 10.1155/2022/1098892 (PMC8800603; doi:10.1155/2022/1098892)
Supplement: Supplementary Materials — Figure S1: α diversity (Shannon index) of the gene, genus, KEGG orthologs (KO), and eggNOG (OG) functions. Figure S2: comparison of abundance of Bacteroidetes in high-fat and low-fat pigs. Supplementary Table S1-S2: sequences of the primers used in this study. [file 1098892.f1.docx]

Figure S1: α diversity (Shannon index) of the gene, genus, KEGG orthologs (KO), and eggNOG (OG) functions in the colon of the high- and low-fat pigs. Data are shown as box plots. The horizontal lines indicate the medians, and the whiskers indicate the lowest and highest points within 1.5× the interquartile ranges into the lower and upper quartiles. H, the high-fat pigs; L, the low-fat pigs.

Figure S2: Comparison of abundance of Bacteroidetes in high- and low-fat pigs. Data are expressed as mean ± SD (n = 5) and analyzed by the unpaired two-tailed Students’ *t*-test. H, the high-fat pigs; L, the low-fat pigs. **P* < 0.05.

Table S1: Real-Time PCR Primers for Porcine Genes

| Gene | GenBank Accession No. | Primer Sequences (5' to 3') | Size (bp) |
| --- | --- | --- | --- |
| *GAPDH* | AF017079 | GGCAAATTCCACGGCACAGTCA | 82 |
|  |  | CTCGCTCCTGGAAGATGGTGAT |  |
| *18S* | NR_046261 | GCCCTATCAACTTTCGATGGTAGTC | 113 |
|  |  | CCTTGGATGTGGTAGCCGTTTCTCA |  |
| *ACC1* | NM_001114269 | GGAGGAATACCCGTGGGAGTAGT | 105 |
|  |  | CTGCTGGATTATCTTGGCTTCAGA |  |
| *FAS* | NM_001099930 | CCTCCCTCAACTTCCGAGACG | 152 |
|  |  | CGCGGGCACCATTCCCATCA |  |
| *MLXIPL* | XM_003124408 | GTCCGACATCTCCGACACACTCT | 97 |
|  |  | CATGTCAGCATTGCCGACATAG |  |
| *SREBP1* | NM_214157 | GCACTTTCTGACCCGCTTCTTC | 82 |
|  |  | CTGCATGGCAACAGGCACCGA |  |
| *LPL* | NM_214286 | CCCTATACAAGAGGGAACCGGAT | 138 |
|  |  | CCGCCATCCAGTCGATAAACGT |  |
| *FABP4* | NM_001002817 | CCCAACCTGATCATCACTGTGAAT | 89 |
|  |  | CCCAATTTGAAGGCAATCTCAGT |  |
| *PPARγ* | NM_214379 | GTGGAGACCGCCCAGGTTTG | 108 |
|  |  | GGGAGGACTCTGGGTGGTTCA |  |

Table S2: Primers used in the present study.

| Item | Primer Sequences (5' to 3') |
| --- | --- |
| Butyryl-CoA: acetate-CoA transferase | AAGGATCTCGGIRTICAYWSIGARATG |
|  | GAGGTCGTCICKRAAITYIGGRTGNGC |
| Butyrate kinase | TGCTGTWGTTGGWAGAGGYGGA |
|  | GCAACIGCYTTTTGATTTAATGCATGG |
